# Early detection of bovine respiratory disease in pre-weaned dairy calves using sensor based feeding, movement, and social behavioural data

**DOI:** 10.1038/s41598-024-58206-4

**Published:** 2024-04-28

**Authors:** Emily V. Bushby, Matthew Thomas, Jorge A. Vázquez-Diosdado, Francesca Occhiuto, Jasmeet Kaler

**Affiliations:** School of Veterinary Medicine and Science, Sutton Bonington Campus, University of Nottingham, Leicestershire, LE12 5RD UK

**Keywords:** Animal health, Calf, Precision livestock farming, Respiratory disease, Machine learning, Machine learning, Animal behaviour, Animal physiology

## Abstract

Previous research shows that feeding and activity behaviours in combination with machine learning algorithms has the potential to predict the onset of bovine respiratory disease (BRD). This study used 229 novel and previously researched feeding, movement, and social behavioural features with machine learning classification algorithms to predict BRD events in pre-weaned calves. Data for 172 group housed calves were collected using automatic milk feeding machines and ultrawideband location sensors. Health assessments were carried out twice weekly using a modified Wisconsin scoring system and calves were classified as sick if they had a Wisconsin score of five or above and/or a rectal temperature of 39.5 °C or higher. A gradient boosting machine classification algorithm produced moderate to high performance: accuracy (0.773), precision (0.776), sensitivity (0.625), specificity (0.872), and F1-score (0.689). The most important 30 features were 40% feeding, 50% movement, and 10% social behavioural features. Movement behaviours, specifically the distance walked per day, were most important for model prediction, whereas feeding and social features aided in the model’s prediction minimally. These results highlighting the predictive potential in this area but the need for further improvement before behavioural changes can be used to reliably predict the onset of BRD in pre-weaned calves.

## Introduction

Disease and poor health significantly impact all aspects of livestock production from animal welfare to farm economics^1^. Of significant concern, bovine respiratory disease (BRD) is estimated to cost the UK cattle industry an estimated £60–50 million per year, is the most common cause of death and poor performance in calves up to 10 months of age^2,3^, and is known to impact production into adulthood^4–7^. Early detection and diagnosis are key to managing disease spread and reducing the impact on the animal, herd, and farm. Moreover, early diagnosis means any treatment administered is likely to be more effective and reduce antimicrobial usage, an important step in tackling antimicrobial resistance—a critical One Health problem^8,9^. Identification and diagnosis of BRD can be done in multiple ways including infrared thermography^10^, thoracic ultrasonography^11^, or laboratory tests such as PCR using nasal swabs^12,13^. However, the most common way to identify a calf with BRD is by using a clinical scoring system, such as the widely adopted Wisconsin Scoring System^14^. Unfortunately, the multifactorial nature of BRD and often late onset of clinical signs, makes it challenging to prevent and diagnose early using typical monitoring and scoring systems^15,16^. Furthermore, close monitoring of individuals is becoming increasingly difficult within larger modern intensive systems^17^. However, over recent years there has been focus on detection of behavioural changes in livestock using various technologies, such as CCTV cameras and accelerometers^18–23^ however, a tool that is accurate and accessible for farmers to identify BRD is not currently available^23^.

Behavioural changes in response to disease, known as sickness behaviours, can often precede obvious clinical signs and diagnosis^24–29^. Consequently, identifying these changes in behaviour is often vital to detecting a health event early. Although manual checking of livestock by farmers is critical and should not be wholly replaced, technologies have the potential to reduce the subjectivity of manual checking, monitor livestock in a time efficient manner, and identify behavioural changes that may be subtle or easily missed by the stockperson^17,19^. Changes in activity have been identified using wearable accelerometers, with BRD positive calves being generally less active and having longer lying times and bouts^30–32^. Similarly, multiple studies have observed changes in feeding behaviours up to five days before clinical signs of BRD are present^32–36^, or in one case up to 7 days prior^35^. It should be noted that only a small number of feeding variables have been explored: typically feeding speed, total milk consumption, and numbers of rewarded and unrewarded visits to the feeder^37^. Despite milk feeders being used to automatically collect the data, the wealth of data produced by these machines is underutilised as a wider range of feeding behaviour features could be engineered and explored from this data. There is also large potential for these novel features to be used with machine learning algorithms to identify disease onset early^38^. To the authors knowledge, only two published studies have used feeding^39,40^, and three have used feeding and activity-based behaviours^41–43^ with machine learning algorithms to predict BRD in pre-weaned calves. These studies have typically produced moderate to high performance values using a range of machine learning algorithms, consequently, highlight the potential in this area but also the need for improvement and further research to identify how best this type of behavioural data can be utilised in early disease prediction in calves. Furthermore, other types of behaviours that are impacted by poor health and disease are yet to be explored. One example is that animal social behaviours that have been shown to be impacted by disease^44,45^ and can have implications for disease transmission^46,47^.

The aim of this study was to use a range of novel and previously researched behavioural features with machine learning classification algorithms to predict bovine respiratory disease events in pre-weaned calves. We also aimed to identify the most effective features in predicting a sickness event. It was hypothesised that using a combination of feeding, movement, and social features would allow for the identification of calves with BRD. To our knowledge this is the first study to use movement and social behaviours to predict BRD onset, as well as to use the combination of all three feeding, movement, and social features, with the aim to predict cases of bovine respiratory disease in pre-weaned calves.

## Method

This study was approved by the Ethical Committee at the School of Veterinary Medicine and Science, University of Nottingham (reference number: 1481150603) and was performed in accordance with relevant guidelines and regulations of the School of Veterinary Medicine and Science, University of Nottingham. All methods are reported in accordance with ARRIVE guidelines^48^.

### Animals and housing

This study took place at the Centre for Dairy Science and Innovation (CDSI) at the University of Nottingham, UK. Calves were managed according to standard procedures for the University of Nottingham’s CDSI. Within two hours of birth calves are fed four litres of pasteurised colostrum and removed from the dam within four hours. All animals were ear tagged with an RFID ear tag as soon as possible after birth, were vaccinated for respiratory disease at nine days old (Rispoval RS+Pi3 IntraNasal; Zoetis) and disbudded from two weeks old. Throughout the study the calves were checked twice daily by the farm staff. Furthermore, based on daily observational checks and information collected during health scoring (see below), sick calves were treated as required by farm staff. All calves were reared in pairs in a straw bedded pen (paired housing pen: 1.5 × 3.5 m), until six to eight pairs of calves were present, creating a cohort of 12–16 calves, and the youngest individual was a minimum of two weeks old. At this stage the cohort was moved into a larger straw pen as a group (group housing pen: 6 × 10 m) where they remained for up to twelve weeks. Throughout the paired and grouped housing, all calves had ad-lib access to water, concentrates and chopped straw, as well as 10L of milk replacer (Milkivit Energizer ECM, Trouw Nutrition GB) per day from the automatic milk feeder (Forster-Technik, COMPACT smart), allocated via RFID recognition. Each allocation is given at two-hour intervals. Once the allowance has been consumed the feeder will not dispense another allowance for two hours. If the calf does not consume the whole allowance, then it is kept available for the whole two-hour interval before the entitlement restarts at two litres. Regardless of age, from day 36 after moving to the group housing the milk entitlement was reduced by 400 ml per day until day 56 when the calves were completely weaned.

Data for this study was collected between the April 2021 and July 2022 for 11 cohorts of 15–17 calves during the group housing stage. Four calves were removed from the cohorts due to health or management reasons, resulting in data collected for a total of 172 animals. The majority were female Holstein–Friesian calves (168) however, one male Holstein–Friesian, one female Aberdeen Angus × Holstein–Friesian and two male Aberdeen Angus × Holstein–Friesian calves were also included in the study.

### Data collection, pre-processing, and feature engineering

All data pre-processing and analysis was carried out using R software^49^. To allow for acclimatisation to the new environment, the first two days after moving to the group pen were removed. Likewise, to remove any change of behavioural change due to weaning, the weaning and post-weaning period were removed from the dataset. This resulted in 33 days of data per cohort.

### Health assessment

During the group housing, all calves was assessed for bovine respiratory disease twice weekly by one of three scorers, three to four days apart, using a modified version of the Wisconsin health scoring system^14^. For each calf individually, this involved scoring the eye, nose, ear, cough, and rectal temperature from zero to three, before these scores were combined to create a total Wisconsin score for each calf on each scoring day (maximum score of 15). Farm treatment records were used to identify days when the calves had been treated for illness.

All health data was collected for all calves, then to assess the level of information required to accurately label an individual as healthy or sick, we compared three different health outcomes which included increasing amounts of health data:

(i) Wisconsin score (WS): used the Wisconsin health scoring system alone. A calf was identified as sick on that day if they had a Wisconsin score of 5 or above. Calves with Wisconsin scores of less than 5 were classed as healthy.
(ii) Wisconsin score corrected for rectal temperature (WS+RT): used the Wisconsin health scoring system corrected for pyrexia, as defined as a rectal temperature of 39.5 °C or above. A calf was classed as sick if it had a Wisconsin score of 5 or more and/or a rectal temperature of 39.5 °C or above. A calf was classed as healthy if they had a Wisconsin score below 5, and a rectal temperature below 39.5 °C.
(iii) Wisconsin score corrected for rectal temperature and treatment records (WS+RT+T): used the Wisconsin health scoring system, corrected for pyrexia, and treatment as recorded by a member of staff. A calf was classed as sick if it had a Wisconsin score of 5 or more and/or a rectal temperature of 39.5 °C or above, and/or had been treated for illness by a member of staff on that day. A calf was classed as healthy if they had a Wisconsin score below 5, a rectal temperature below 39.5 °C, and had not been treated for illness by the farm staff.

### Feeding behaviours

Feeding data was automatically collected by the computerised milk feeder (Forster-Technik, COMPACT smart). The feeder collects a range of data each time a calf visits the feeder including: calf ID, date, visit start time, visit end time, total milk consumed, drinking speed, actual entitlement at that time (ml), time the next entitlement is due, visit with entitlement, and visit without entitlement (definitions available in the supplementary material). These data were exported from the SD card used to create feeding features (Table 1).

**Table 1:** The 21 core included features and definitions. For each core feature the lags from one to five days, three- and five- day moving averages, and the difference between the present day and the three- and five- day moving averages were also included as delta features. Meals were defined as visits to the feeder by the same calf that were close in time (<120s), including visits when the calf wasn’t entitled to milk or where the calf doesn’t consume any milk.

Feature	Definition
Feeding	
Entitled meals	The daily number of entitled meals per calf, calculated by the visits to the feeder by the same calf that were close in time (< 120 s) when the calf is entitled to milk
Unentitled meals	The daily number of unentitled meals per calf, calculated by number of visits to the feeder by the same calf that were close in time (< 120 s) when the calf is not entitled to milk
Visits within an entitled meal	The number of visits per day by each calf when an entitlement was due/present
Nutritive meals	Visits to the feeder by the same calf that were close in time (< 120 s) when the calf is entitled to and consumes milk
Entitled meal interval standard deviation	The latency between the end of the last meal with entitlement and the following meal with entitlement (s). The standard deviation of the mean per day for each calf
Entitled meal duration mean	The mean entitled meal duration per calf per day, as defined as the time spent at the feeder during a meal when the calf was entitled to milk (s)
Entitled meal duration minimum	The minimum entitled meal duration per calf per day, as calculated by the time spent at the feeder during a meal when the calf was entitled to milk (s)
Entitled meal duration standard deviation	The standard deviation of the mean entitled meal duration per calf per day, as defined as the time spent at the feeder during a meal when the calf was entitled to milk (s)
Unentitled meal interval mean	The daily mean for each calf for the interval between unentitled meals
Unentitled meal duration mean	The daily mean for each calf for the duration of unentitled meals
Feeding speed	Mean daily feeding rate calculated by the feeder for each visit to the feeder where the calf is entitled to and consumes milk
Entitled latency mean	The mean per day per calf for the time between the entitlement release and the calf’s first visit to the feeder after the new entitlement is available (s)
Latency after previous calf mean	The daily mean latency for the calf to enter the feeder after the previous calf as defined by the latency between one calf finishing a visit and a second calf entering the feeder
Latency after previous calf minimum	The daily minimum latency for the calf to enter the feeder after the previous calf as defined by the latency between one calf finishing a visit and a second calf entering the feeder
Times latency under 60 s	Number of visits per day where the calf entered the feeder within 60 s of the previous calf leaving
Movement	
Walking distance	The daily distance travelled per calf, computed as the sum of the distance between consecutive points over 24 h for each day using the coordinates reported by each calf’s location tag
Mean turning angle correlation	Mean turn angle autocorrelation was computed as the inverse of angular autocorrelation, where angular autocorrelation was the sum of squares of chord distances between N successive turn angles ρ^53^
Mean residency time	The length of time an individual spends stationary inside a circle of a 1-m radius centred around its location, without leaving it for more than 1 min, averaged over 24 h to create a daily measure
Daily sinuosity	Sinuosity represents the level of turning in the movement path and it is used to reliably estimate the tortuosity of a random search path. Sinuosity is a function of both the mean cosine of turning angles and step length and varies between 0 and 1 ^54,55^
Social	
Mean contact duration	The mean duration of social proximity interactions defined as two calves being within less than one meter of distance for a minimum of three minutes ^44^
Number of encounters	Number of social proximity interaction encounters as defined by two calves being within less than one meter of distance for a minimum of three minutes ^44^

### Movement behaviours and social interactions

The following movement features: walking distance, mean turning angle autocorrelation, mean residency time and sinuosity, as described in Table 1, were calculated using location data from ultrawideband sensors^50^ mounted to a collar worn by the calves. Location sensors recorded the relative (x, y) coordinates of the animal and were set to a frequency of 1 Hz. Location data was processed and filtered as in Occhiuto, et al.^51^.

The following social features: mean contact duration and number of proximity encounters, as described in Table 1, were computed using the location data from the ultrawideband sensors worn by the calves. For the social features interactions were defined as “*proximity interactions below a threshold distance for a minimum duration of time between a pair of calves*”^44^. A 1-m spatial threshold and 3-min threshold were used for the duration as in Vázquez-Diosdado, et al.^44^.

Full details of how movement and social interaction data were collected and pre-processed can be found in Occhiuto, et al.^51^ and Vázquez-Diosdado, et al.^44^ respectively.

### Feature reduction

All 47 features (39 feeding, 4 movement, and 4 social features) were assessed for correlations and any two features with a correlation above 0.7 or below − 0.7 were removed based on how often they correlate with other features (i.e. if one feature correlates with lots of features it was removed instead of the feature that didn’t correlate with as many) and relevance based on previous literature (e.g. feed speed has been shown to be important in predicting heath^34,35,52^ and would be retained over another feature with unknown importance). This resulted a total of 21 core features (Table 1). For all 21 core features, lags from one to five days, three- and five- day moving averages, and the difference between the present day and the three- and five- day moving averages were calculated as delta features. This resulted in a total of 229 features in total.

### Classification algorithms

To find the best machine learning algorithm, three supervised learning algorithms were selected for comparison: random forest, elastic net, and gradient boosting machine. All three were employed using the caret package in R^56^, and each health outcome dataset was balanced into a 40% sick and 60% healthy split. All models were implemented using each of the three outcomes resulting in a total of nine classification models, with each one implemented as follows. Firstly, the optimal hyperparameters for each model type were found using an 80:20 data split for training and testing, respectively. For gradient boosting machine this was the number of trees, the number of splits in each tree, learning rate, and the minimum number of observations in the trees terminal node. Hyperparameters for random forest were the number of variables to randomly sample at each split, the maximum number of terminal nodes, and the minimum node size. Alpha and lambda were optimised for elastic net. Optimal parameters were obtained using a grid search through a set of varying parameters and the final parameters used can be found in the supplementary material.

Secondly, all models were implemented with the full 229 features and the 40% sick 60% healthy balanced dataset using a fivefold cross validation technique. Algorithm performance was evaluated using the following performance metrics: accuracy, specificity, sensitivity, precision, F1-score. Feature importance was calculated using the *varImp* function from the caret package^56^. Finally, to find the optimal number of features, the feature ranking was used to implement the classification algorithm using an increased number of features added according to their importance. The performance for each model with the number of included features was computed and the final optimal number of features selected based on overall performance. The best model was selected based on the F1-score and sensitivity performance metrics, as they are deemed most important to predict the true positives of the disease. To enable fair comparison, the final reduced feature number was kept the same for all models. To calculate the feature stability, the model was run for 100 iterations and the feature importance for all features was calculated for each iteration using the *varImp* function.

### Ethical approval

This study was approved by the Ethical Committee at the School of Veterinary Medicine and Science, University of Nottingham (reference number: 1481150603).

## Results

Of the 172 calves included in the study 61, 104, and 123 got sick as defined by outcomes one, two, and three respectively (for detail, please see Table S3 in the supplementary material).

### Model comparison and optimal feature number

**Figure 1:**
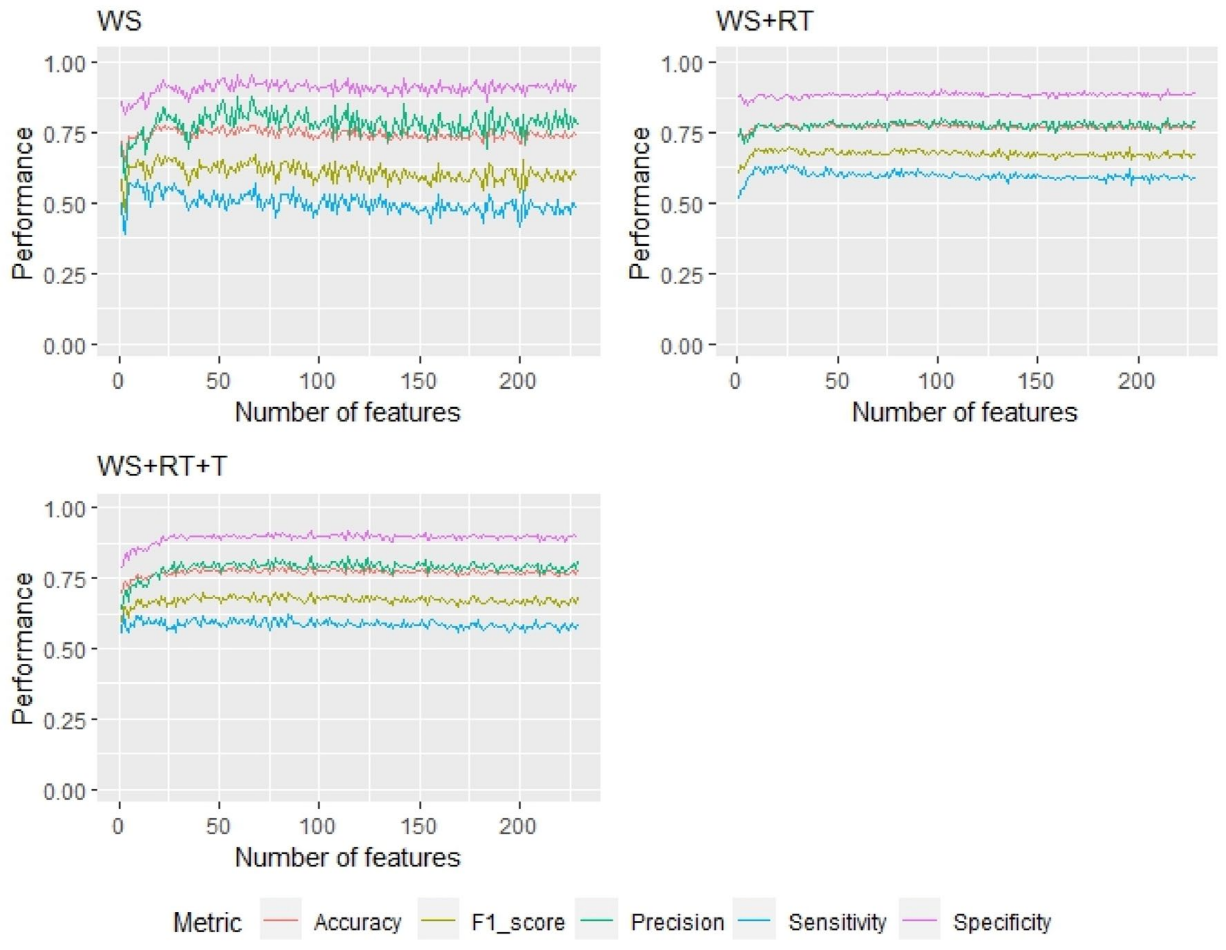
Gradient boosting machine model performance for each health outcome with increasing number of features included based on their importance (calculated using the *varImp* function).

**Figure 2:**
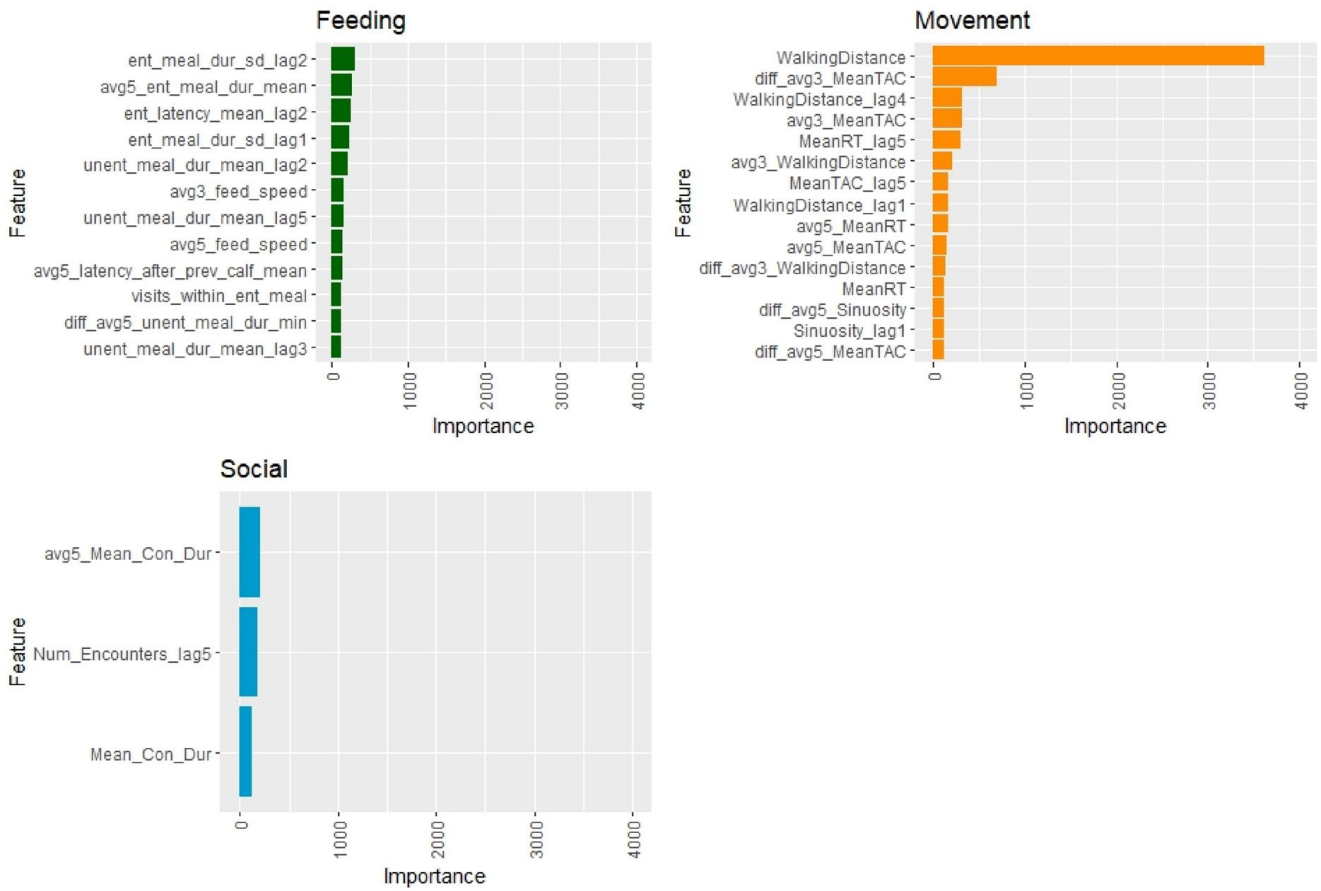
Feature importance (calculated using the *varImp* function) for the final 30 features included in the gradient boosting machine WS+RT outcome. In the y-axis “avg5” and “avg3” indicates five- and three-day moving averages, “diff_avg5” and “diff_avg3” indicates the difference between the current day and the moving average, and lag1-5 indicates a lag of up to five days.

Overall, the optimal number of features across all three model types was determined to be 30 (Figs. 1 and 2). Of the three classification algorithms, the gradient boosting machine performed best with the final 30 features and therefore for simplicity will be the focus of the results and discussion. Full results of the elastic net and random forest models can be found in the supplementary material (Table S4).

### Model performance

Using a gradient boosting machine classification algorithm, outcome WS+RT (Wisconsin score corrected for pyrexia) with both 229 and 30 features, the classification algorithm outperformed both outcome WS and WS+RT+T on F1-score and sensitivity. Additionally, using the WS+RT outcome with 30 features the performance of the gradient boosting machine classification algorithm had the highest F1-score and sensitivity (Table 2). However, outcome WS and WS+RT+T produced higher specificity, precision, and in the case of WS+RT+T, higher accuracy than the WS+RT outcome.

**Table 2:** Performance metrics for the gradient boosting machine algorithm for all three health outcomes and for both all 229 and the model including only the 30 features ranked as most important. The highest value of each row is highlighted in bold and standard deviation in brackets.

	Health assessment		
	WS	WS+RT	WS+RT+T
**229 Features**			
Accuracy	0.738 (± 0.063)	0.764 (± 0.072)	**0.765** (± 0.038)
Precision	**0.804** (± 0.161)	0.775 (± 0.116)	0.785 (± 0.128)
Sensitivity	0.458 (± 0.168)	**0.580** (± 0.127)	0.570 (± 0.049)
Specificity	**0.925** (± 0.084)	0.887 (± 0.076)	0.896 (± 0.029)
F1 score	0.584 (± 0.097)	**0.663** (± 0.100)	0.660 (± 0.071)
**30 Features**			
Accuracy	0.761 (± 0.098)	0.773 (± 0.072)	**0.774** (± 0.047)
Precision	0.795 (± 0.153)	0.766 (± 0.139)	**0.797** (± 0.162)
Sensitivity	0.541 (± 0.186)	**0.625** (± 0.127)	0.585 (± 0.091)
Specificity	**0.907** (± 0.072)	0.872 (± 0.098)	0.896 (± 0.043)
F1 score	0.644 (± 0.135)	**0.689** (± 0.084)	0.672 (± 0.113)

### Feature importance

Before reduction, the original 229 features were made up of 168 feeding features (73.79%) 38 movement features (17.46%), and 20 social features (8.73%). In contrast, in the final dataset of 30 features, movement features made up the highest proportion with 15 features (50%), feeding with 12 features (40%), and social features with 3 (10%) as shown in Fig. 2.

### Feature stability

**Figure 3:**
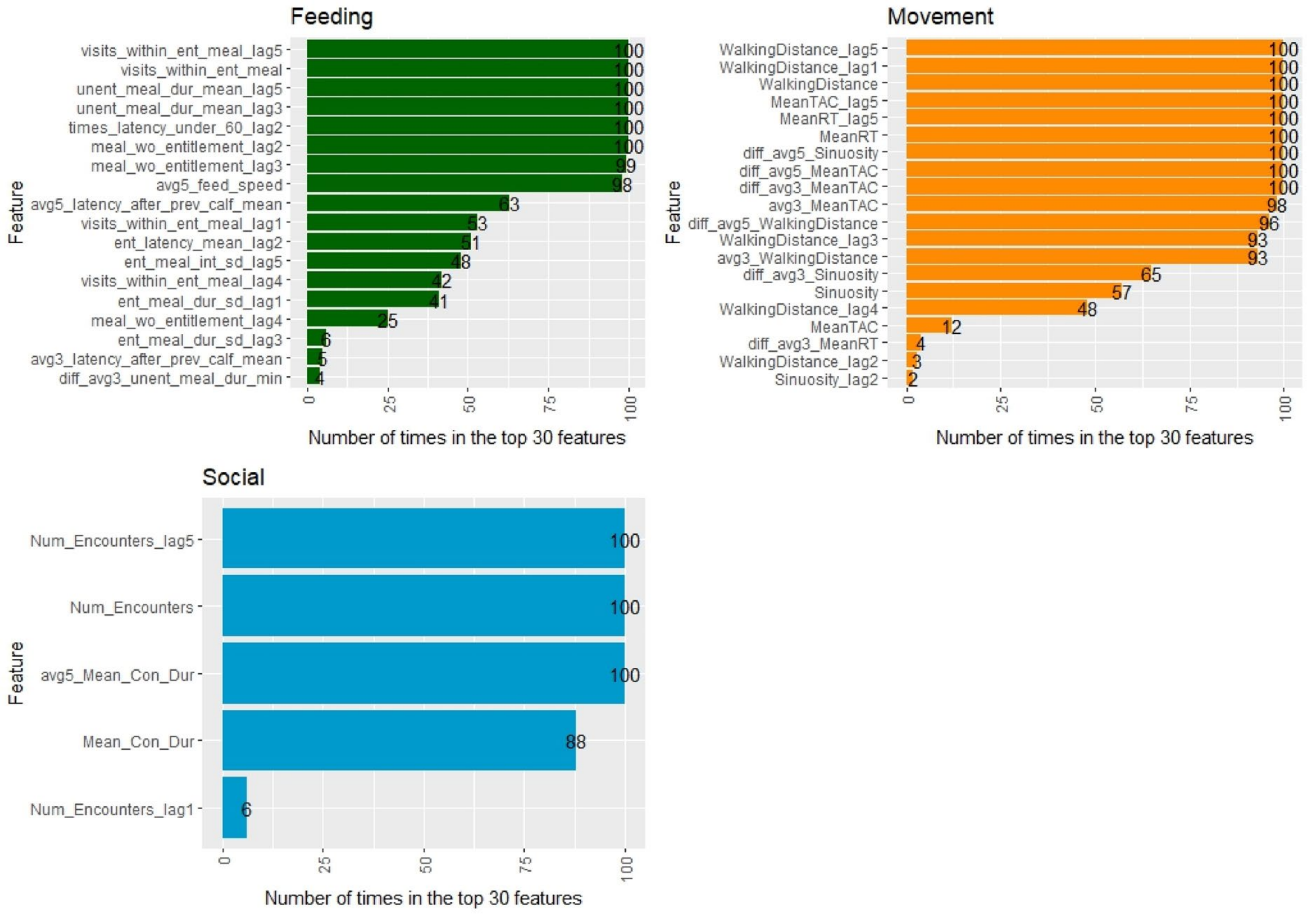
The 43 features that were in the top 30 most important features (as determined by the *varImp* function) across 100 iterations of the model. The bar numbers indicate the number of times out of the 100 iterations the feature was included in the top 30 most important.

Across the 100 iterations, 43 features were in the top 30 most important for at least one of these iterations, and 18 were always in the top 30 (Fig. 3). The other 186 features were never ranked as one of the 30 most important. Walking distance and mean turning angle autocorrelation difference between the current day and the moving average were always ranked one and two, respectively.

## Discussion

To the authors knowledge, this is the first study to explore how social and movement behaviours in combination with feeding behaviours, can be used with machine learning algorithms to predict bovine respiratory disease in pre-weaned calves. Identifying bovine respiratory disease early is key to the treatment and management of the disease, and previous research has shown that changes in behaviour may be indicative of disease status before clinical symptoms are evident^26,29^. It has previously been identified that BRD typically results in a decrease in activity^31,33^ and change in feeding patterns^37^. In this study, walking distance was by far the feature that contributed to the model most significantly and was consistently ranked as the top feature (Fig. 3). Furthermore, five of the top 30 features were variations of walking distance, showing that this measure of movement was key in the prediction of respiratory disease in the calves in this study. This indicates that the daily distance travelled by the calf when sick was likely to be greatly reduced and is in line with previous research showing that measures of activity, such as lying time^32^ and the number of steps taken daily^57^, to be of high importance in the early detection of respiratory disease. Similarly, 50% of the final dataset included features were movement based, and when exploring the feature stability, half of the 18 features always ranked in the top 30 after 100 iterations were movement features. This highlights that movement behaviours, specifically walking distance and the difference between the day and 3 day moving average of the mean turning angle correlation (Figs. 2 and 3), were where the largest behavioural changes occurred in BRD positive calves.

The importance of movement behaviour in predicting BRD in this study is in contrast with the focus on feeding behaviours in previous literature^32–34,37^. Previously studies have used a small number of feeding behaviour features^39,40^, and therefore we aimed to explore the possibility of using automatic milk feeding machines to create novel features to predict a health event. We not only included commonly employed feeding variables, but we engineered new ones, such as the latency to enter the feeder after the previous calf, among others, to ensure all aspects of this behaviour were assessed. Of the 21 core features (i.e. before delta versions; Table 1), 13 were novel for this problem and of the final 30 top features (Fig. 2), 18 were based on these novel features. Included in the features that were most important in the model were features, or variations of features that have been explored in previous studies, including feeding speed (two features), unentitled meal duration (four features), entitled meal duration (four features), and visits to the feeder within an entitled meal. These results agree with previous studies that have shown a reduction in feeding speed^34,35^ and unrewarded visits^35,58^ to the feeder when calves are sick, indicating that they are still motivated to visit the feeder but only as much as is necessary. However, despite the focus on feeding behaviours in the previous literature and the inclusion of novel feeding variables in the present study, changes in feeding behaviour contributed minimally to BRD prediction (Fig. 2). This contrasts with two previous studies that only used feeding behaviour data from automatic milk feeders. Ghaffari, et al.^40^ demonstrated high performance values (sensitivity: 0.826; specificity: 0.790; accuracy: 0.800; negative predictive value: 0.970) except for the positive predictive value (0.365) using a convolutional neural network approach. Moreover, a recent study achieved high precision (0.814), recall (0.740), and F1-score (0.775)^39^ using a gradient boosting machine algorithm, using a different dataset the performance was much lower (precision: 0.484; recall: 0.219; F1-score: 0.301), and again performance varied greatly when using random forest or generalised linear models. Differences in how a calf is classed as sick may explain some of the difference in results between the current and previous research as both previous papers used more frequent health scoring and lower Wisconsin score thresholds^39,40^. The variability in performance found by Perttu, et al.^39^ and the results of the present study may also indicate that feeding is an essential behaviour which during disease is altered but otherwise maintained^59^, consequently explaining why feeding behaviours is not always a good predictor of BRD, or disease, status in calves. In contrast social behaviours are less likely to be essential, and disease has been shown to result in a higher level of social isolation, or a reduction in social interactions, in both cattle and other species^60,61^. Unlike previous research in this area^41–43^, we also included social behavioural features—the daily number of encounters and the contact duration. As expected, the model used the contact duration and number of encounters (social features) to determine if a calf was BRD positive (Fig. 2). The average daily duration of contact over the previous five days and the number of encounters on the day and lagged for five days were consistently ranked in the most important 30 features for 100 iterations of the model. However, like the feeding behaviours, the social features only contributed minimally to the model prediction in comparison to walking distance and other movement behaviours (Fig. 2). As only two types of measures of social behaviour were explored here, future research should aim to expand on this to enable a better understanding of how other social metrics, such as social ranking and number of displacements, may be used to predict bovine respiratory disease.

Overall, a gradient boosting machine algorithm produced the highest and most consistent performance throughout, with moderate values for all performance (accuracy: 0.773; precision: 0.766; sensitivity: 0.625; F1-score: 0.689), except specificity which was higher (0.872; Table 2). Performance metrics for the gradient boosting machine algorithm for all three health outcomes and for both all 229 and the model including only the 30 features ranked as most important.. Previously when focusing on a moving average and random forest combined model, Bowen and colleagues’^43^ also achieved a similar accuracy (0.75), but higher specificity (0.95) and lower sensitivity (0.54) than this study. The accuracy achieved in the present study (0.773) is like that attained by Casella, et al.^41^ who reported an accuracy of between 0.70 and 0.90 depending on the proposed budget. In contrast our model performance was much lower than that found by Cantor, et al.^42^ who achieved accuracy, precision, recall and F1-score values of 0.99 each. Although, it should be noted that Casella, et al.^41^ and Cantor, et al.^42^ included the individual score for each area (nose, eye, cough etc.) as features in the model, which is likely to be beneficial in the prediction of sick calves labelled as such based on this scoring system. Furthermore, Casella, et al.^41^ used different ways of categorising and labelling the data which makes it difficult to compare with the present study. However, allowing for more nuance to be recognised rather than having binary healthy and sick categories may aid in identifying sick calves and this would be beneficial to explore in future research. In previous literature the identification and thresholds for a calf to be classed as BRD positive or sick vary greatly, highlighting the difficulty of identifying BRD and the need for more understanding of the most accurate way to do so. In this study we compared three different methods of classifying a calf as BRD positive and resulted showed that using the Wisconsin scoring system corrected for high rectal temperature (WS+RT) resulted in the highest model performance, indicating that this was the best way to label a calf as BRD positive of the three methods compared here. When selecting the best outcome, sensitivity was prioritised as when trying to predict disease onset as it is critical that true positives are not missed. Outcome WS+RT produced the highest sensitivity but this was, however, still a moderate value (0.625), highlighting the need for further investigation to improve this area. Although accuracy was higher for the outcome WS+RT+T (WS: 0.761; WS+RT: 0.773; WS+RT+T: 0.774), both outcomes WS and WS+RT+T produced higher specificity (WS: 0.907; WS+RT: 0.872; WS+RT+T: 0.896), and precision (WS: 0.795; WS+RT: 0.766; WS+RT+T: 0.797), the higher F1-score achieved by the WS+RT outcome (WS: 0.644; WS+RT: 0.689; WS+RT+T: 0.672) shows its better overall balance of performance (Table 2).

BRD can present with a range of symptoms in varying severity^16,62^, therefore it is possible that the correction for a high temperature ensures that calves with BRD but with few clinical signs were identified correctly as sick. In contrast, correcting for both rectal temperature and treatment reduced model performance in comparison to correcting for a high temperature alone. As calves were only treated by the farm staff if they presented as clinically sick, this was unexpected, but may be because we do not have data to confirm why the calf was treated. This limitation may have introduced calves to the dataset that may have other illness, not just BRD, and therefore would likely have different behavioural patterns. This was previously demonstrated by Knauer, et al. (2017) who showed that feeding behaviours were different depending on the type of illness^35^. In this study, using the Wisconsin scoring system alone produced the worst model performance suggesting that this is not the best way to identify and diagnose BRD positive calves. The Wisconsin scoring system, where a score of 5 or above is considered to indicate BRD, is widely adopted within the scientific literature^14,32,63^, but has been shown to be moderately accurate at identifying BRD^64^. It should be noted that due to the practicality of long-term data collection, in this study the Wisconsin scoring was carried out twice weekly rather than daily as done in other studies^31–33^. Daily health assessment may improve the performance by ensuring the ground truth data was as specific to the data as possible. To further improve accuracy of identifying calves with BRD, there is some evidence to show that combining Wisconsin scoring with thoracic ultrasound scoring can improve the sensitivity of BRD identification and enables the identification of subclinical BRD^11,64,65^. Moreover, including objective measures of BRD, such as laboratory diagnostic tests which are typically more accurate than clinical scoring systems^12^ , would aid the development of precision livestock tools ^66^. It is also important to note that the prevalence of BRD in this study population will be different to other calf populations, which will invariably impact performance outcomes and may explain some variation between studies.

In conclusion, the combination of social, feeding, and movement behaviours collected using automatic milk feeders and location data have the potential to predict BRD in pre-weaned calves. To the authors knowledge, this is the first study to include social and movement features with machine learning algorithms to predict BRD status in pre-weaned calves. Changes in movement behaviours, as measured by the distance walked per day, was the best indicator is a change in disease status and in contrast feeding and social features only aided in the model’s prediction minimally. Overall, model performance was moderate to high, highlighting the predictive potential in this area but the need for further improvement before behavioural changes can be used to reliably predict BRD. Exploration into other behavioural changes is required to understand further how behavioural change pre-clinical signs may be used to inform BRD status in pre-weaned calves.

### Supplementary Information

Supplementary Tables.

## Data Availability

The data used for the analysis in this paper will be available on reasonable request from the author.
