# Supplementary material for: Early detection of bovine respiratory disease in pre-weaned dairy calves using sensor based feeding, movement, and social behavioural data
Source: Sci Rep. 2024 Apr 28;14:9737. doi: 10.1038/s41598-024-58206-4 (PMC11056383; doi:10.1038/s41598-024-58206-4)
Supplement: Supplementary file 1 — Supplementary Tables. [file 41598_2024_58206_MOESM1_ESM.pdf]

Supplementary Material

Table S1. Automatic milk feeder data definitions

	**Definition**
**Start Visit**	The time a calf starts a visit to the feeder
**End Visit**	Timestamp for when the calf exits the feeder after each visit
**Actual entitlement**	Entitlement at that time (ml)
**Visits With Entitlement**	The number of visits per day by each calf when an entitlement was due/present.
**Visits Without Entitlement**	The number of visits to the feeder each day by each calf when an entitlement wasn't due/present.
**Feeding Speed**	Mean daily feeding rate for each visit to the feeder where the calf is entitled to and consumes milk.
**Total Milk**	The total amount of milk consumed by each calf for the entirety of the calf's life or time with that machine.

Table S2. Optimal parameters used for each model type and health assessment outcome.

**Model Type**	**WS**	**WS+RT**	**WS+RT+T**
**Random Forest**	Mtry = 4<br>Maxnode = 10<br>Ntrees = 700	Mtry = 9<br>Maxnode = 11<br>Ntrees = 950	Mtry = 10<br>Maxnode = 9<br>Ntrees = 550
**Elastic Net**	Alpha = 0<br>Lambda = 3.939455	Alpha = 0<br>Lambda = 1.616245	Alpha = 1<br>Lambda = 0.0001
**Gradient Boosting Machine**	n.Trees = 400<br>Interaction.depth = 5<br>Shrinkage = 0.05<br>n.Minobsinnode = 3	n.Trees = 500<br>Interaction.depth = 3<br>Shrinkage = 0.005<br>n.Minobsinnode = 3	n.Trees = 350<br>Interaction.depth = 7<br>Shrinkage = 0.005<br>n.Minobsinnode = 5

Table S3. Descriptive table to show the number of calves that experienced one or more BRD sick classification per health assessment outcome.

	WS	WS+RT	WS+RT+T
**Total number of sick calves**	61	104	123
**No. calves with one sick event**	37	51	36
**No. calves with two sick events**	10	27	32
**No. calves with three sick events**	11	16	29
**No. calves with four sick events**	1	7	13
**No. calves with five sick events or more**	2	3	13

Table S4. Results for random forest and elastic net classification algorithms with all 229 features.

	WS	WS+RT	WS+RT+T
**Random Forest**			
Accuracy	0.766	0.752	0.724
Precision	0.916	0.962	0.903
Sensitivity	0.458	0.396	0.348
Specificity	0.972	0.989	0.975
F1 Score	0.611	0.562	0.502
**Elastic Net**			
Accuracy	0.655	0.636	0.670
Precision	0.727	0.611	0.584
Sensitivity	0.222	0.251	0.614
Specificity	0.944	0.892	0.707
F1 Score	0.340	0.356	0.599
